# Value of dual-energy computed tomography in the diagnosis of bowel ischemia in patients with mechanical small-bowel obstruction: a retrospective, dual-center study

**DOI:** 10.1007/s00330-025-11635-9

**Published:** 2025-05-06

**Authors:** Sébastien Mulé, Baptiste Brault, Maxime Blain, Nada Neifar, Caroline Touloupas, Isabelle Boulay-Coletta, Edouard Reizine, Alain Luciani, Marc Zins

**Affiliations:** 1 Medical Imaging Department, AP-HP, Henri Mondor University Hospital, 1 rue Gustave Eiffel, 94000 Créteil, France; 2 Faculty of Health, University of Paris Est Créteil, Créteil, France; 3 INSERM IMRB, U 955, Team 18, Créteil, France; 4 Department of Medical Imaging, Paris Saint Joseph Hospital, 185 rue Raymond Losserand, 75014 Paris, France

**Keywords:** Dual-energy CT, Small-bowel obstruction, Bowel ischemia, Iodine

## Abstract

**Objectives:**

To investigate the diagnostic value of rapid-kV-switching dual-energy CT (DECT) for identifying bowel ischemia in patients with mechanical small-bowel obstruction (SBO), compared to 120 kVp-equivalent CT.

**Materials and methods:**

This retrospective dual-center study included 112 patients with mechanical SBO. Clinical and surgical outcomes with histological findings were recorded as the reference standard. Three readers independently reviewed true unenhanced (TUE) and portal-venous 77-keV virtual monoenergetic images (VMI) (dataset#1), virtual unenhanced (VUE), iodine, and 50-keV VMI (dataset#2). Ischemia was defined as the presence of at least two CT features among reduced bowel-wall enhancement, diffuse mesenteric haziness, and a closed-loop mechanism. Unenhanced bowel-wall attenuation was also analysed. Bowel-wall attenuation and iodine concentration were measured in involved loops and proximal dilated loops. Sensitivity and specificity were calculated for each CT feature. Association between iodine concentration and bowel ischemia was analysed using ROC curves.

**Results:**

Forty-one (37%) patients underwent surgery. Twenty-four (21%) patients had findings of bowel ischemia, including 11 (10%) patients with irreversible ischemia (necrosis). Diagnostic performance for ischemia and necrosis was similar between both datasets for all readers (*p* > 0.49 for ischemia and *p* = 1 for necrosis). Increased bowel-wall attenuation on VUE or TUE images had a non-significantly different diagnostic value for all readers (*p* > 0.21 for ischemia and *p* > 0.78 for necrosis). Bowel-wall iodine concentration identified bowel necrosis with 82% sensitivity and 83% specificity (optimal cutoff value 1.82 mg/mL).

**Conclusion:**

DECT performed similarly to 120 kVp-equivalent CT for the diagnosis of bowel ischemia in patients with mechanical SBO. VUE images offer a viable alternative to TUE images for the identification of increased bowel-wall attenuation. Bowel-wall iodine concentration accurately identifies bowel necrosis.

**Key Points:**

***Question*** *Dual-energy CT (DECT) could detect small-bowel ischemia caused by mechanical small-bowel obstruction (SBO), but its performance is unknown*.

***Findings**** Reduced bowel-wall enhancement and increased unenhanced bowel-wall attenuation were not significantly different between DECT and 120 kVp-equivalent CT. Bowel-wall iodine concentration identified necrosis with 82% sensitivity and 83% specificity*.

***Clinical relevance*** *DECT performed similarly to 120 kVp-equivalent CT in depicting bowel ischemia in patients with mechanical SBO, while adding quantitative analysis of bowel-wall iodine concentration may further help identify patients requiring surgery*.

**Graphical Abstract:**

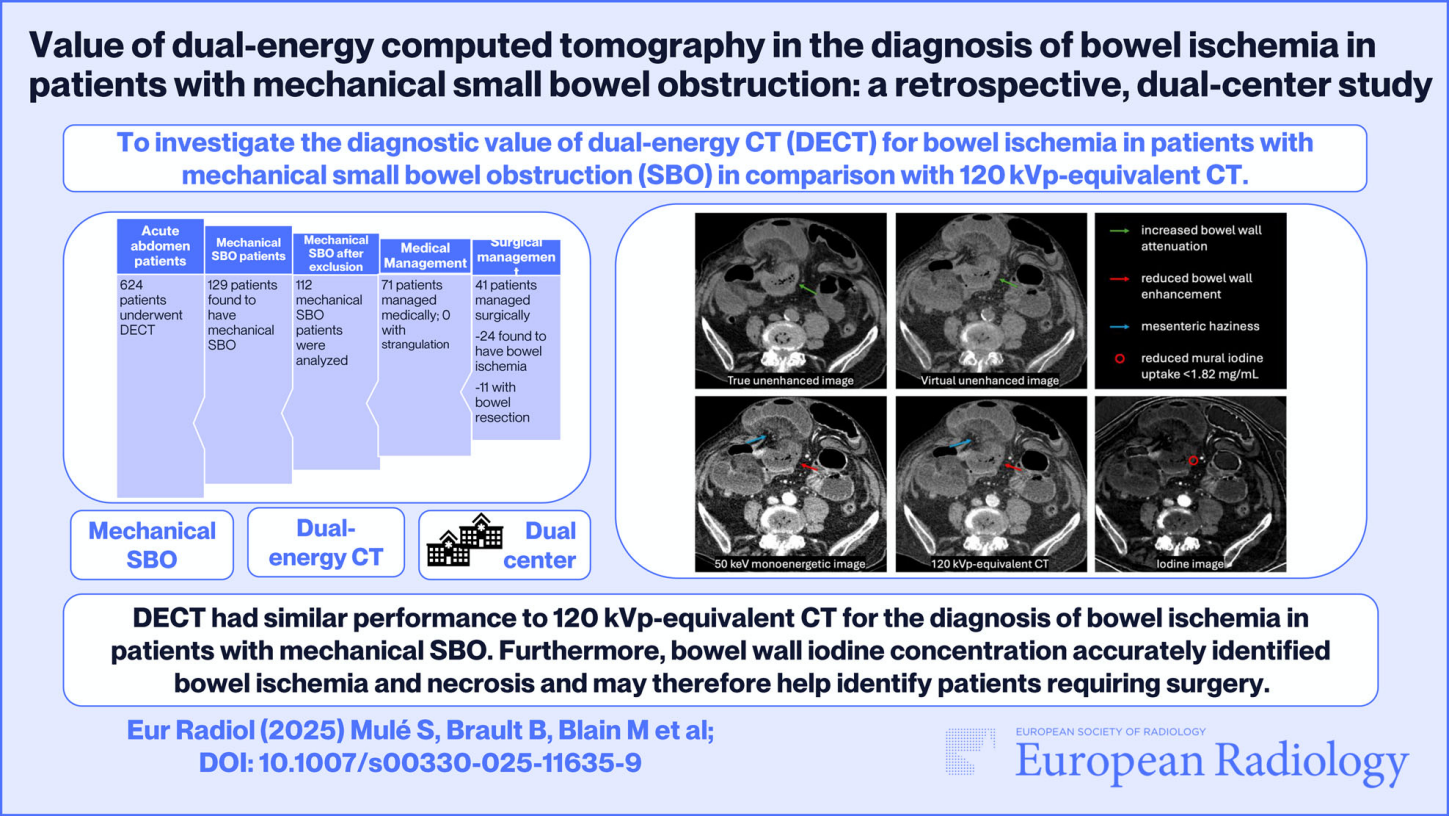

## Introduction

Contrast-enhanced multidetector CT (CECT) is the reference standard for the evaluation of small-bowel obstruction (SBO), a frequent cause of acute abdomen with potential life-threatening complications. Despite the high accuracy of CECT for its diagnosis and location, the management of SBO using imaging data remains challenging. For instance, the early identification of patients presenting with SBO and requiring surgery is critical [1, 2]. Notably, being able to accurately diagnose small-bowel ischemia is of paramount importance [3]. Indeed, ischemia occurs in about 10% of all cases of SBO and is associated with poor prognosis and increased mortality [4, 5]. The combination of 3 CT findings, including reduced bowel wall enhancement, a closed-loop mechanism, and diffuse mesenteric haziness, has been reported to accurately identify ischemia in adhesive SBO with very high specificity; as a result, conservative management may be sufficient until at least two of these three findings are present [6]. Ischemia may lead to irreversible transmural bowel necrosis, a life-threatening complication requiring prompt surgical resection of necrotic bowel segments. Again, CT plays a pivotal role in identifying bowel necrosis. Increased attenuation of bowel wall on unenhanced CT acquisitions predicts bowel necrosis with high specificity [7–10]. In addition, the acquisition of unenhanced images has been reported to improve the performance and the reproducibility in evaluation of the bowel wall enhancement and thus the diagnosis of ischemia [11].

However, the acquisition of unenhanced images in addition to contrast-enhanced images increases the radiation dose to patients. Moreover, the sensitivity of polychromatic multidetector CT for depicting reduced or absent segmental bowel wall enhancement remains moderate with substantial interreader variability [3, 7, 8, 11, 12]. In that context, dual-energy CT (DECT) appears as a very attractive imaging modality in patients with SBO. DECT allows the reconstruction of low keV virtual monoenergetic images (VMI) that may increase the difference in attenuation between ischemic and non-ischemic bowel segments and thus may improve the detection of ischemia [13, 14]. DECT also characterizes materials based on their differential attenuation profile as a function of energy level, with two main potential advantages in the setting of SBO: (i) virtual unenhanced (VUE) images may serve as a reference for bowel wall attenuation and thus replace true unenhanced (TUE) images, and (ii) iodine-specific images may help improve the qualitative evaluation of bowel wall enhancement but also provide an additional quantitative biomarker of ischemia [13, 15].

The aim of this bicentric study was to investigate the diagnostic value of rapid kV-switching single-source DECT for the detection of small-bowel ischemia (including reversible ischemia and necrosis) in patients with mechanical SBO in comparison with 120 kVp-equivalent CT. Notably, the performance of monoenergetic images at 50 keV for the identification of reduced bowel wall enhancement was compared to that of monoenergetic images at 77 keV (considered as equivalent to conventional single-energy CT images obtained at 120 kVp). In addition, the diagnostic value of VUE images for the identification of increased bowel wall attenuation was compared to that of TUE images.

## Materials and Methods

### Study participants

The institutional review boards (IRB) of the two study centers approved this retrospective, observational study and waived the informed consent requirement (CRM-2209-296 and CRM-2404-403).

**Fig. 1:**
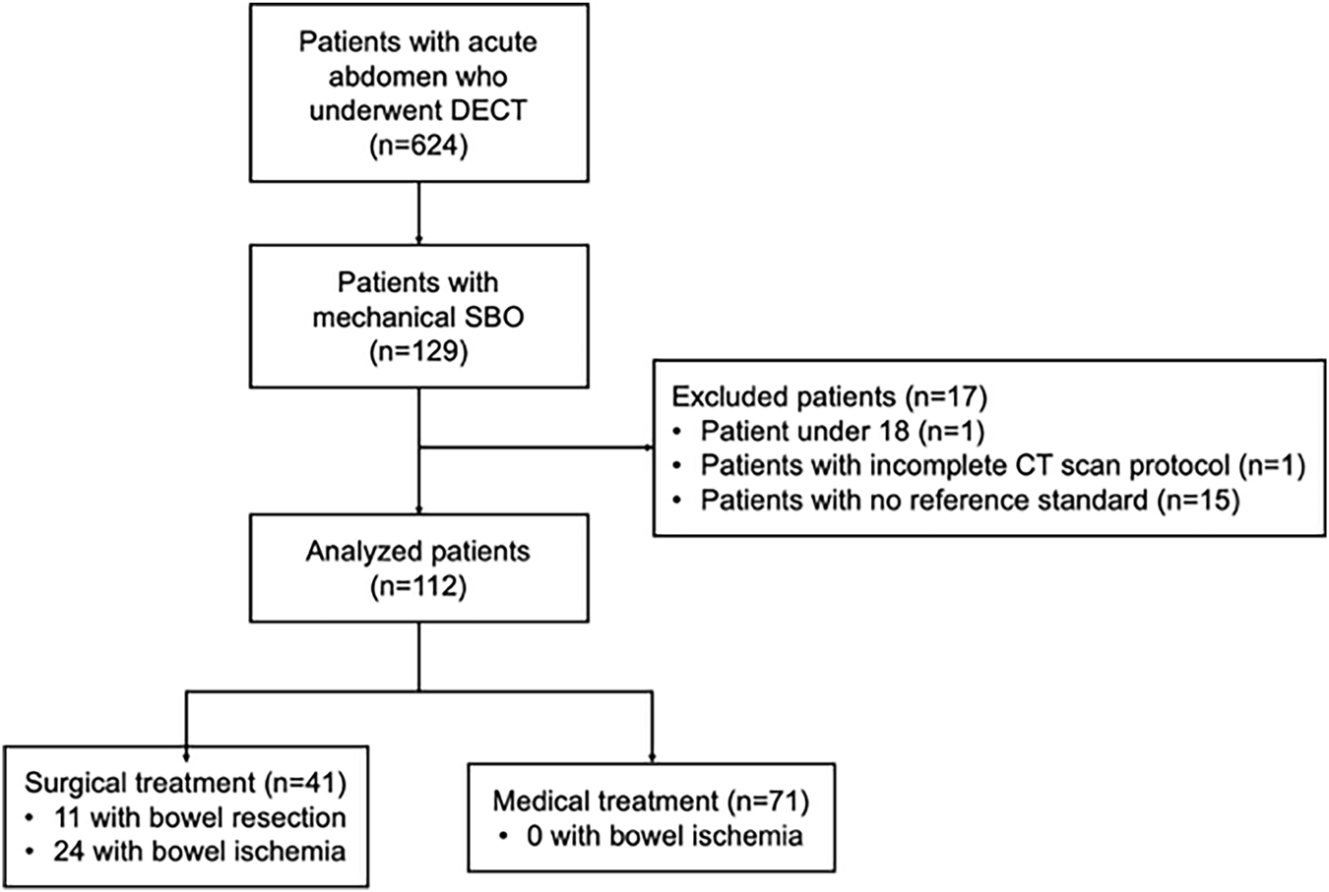
Patient flowchart. DECT, Dual-energy CT; SBO, small-bowel obstruction

Patients who underwent contrast-enhanced DECT for acute abdomen between November 2019 and September 2022 at Henri Mondor University hospital (AP-HP, France) or at Paris-Saint-Joseph hospital (Paris, France) were considered for inclusion (Fig. 1). Patients were included if they had a mechanical SBO at DECT. Exclusion criteria were patients under 18, incomplete CT scan protocol (absence of TUE or portal-venous phase), and absence of reference standard. A total of 112 patients (median age, 71 years; interquartile range (IQR), 60–84 years) were included. Recorded demographic characteristics and clinical data included age, sex, body mass index (BMI), cause and mechanism of mechanical SBO, serum C-reactive protein (CRP) level, white blood cell count, serum creatinine level, serum hematocrit level, and outcome (medical and/or surgical management, death).

### Reference Standard

Bowel ischemia (including both reversible ischemia and necrosis) was diagnosed surgically in all patients and confirmed histologically in those patients who underwent bowel resection. Bowel necrosis was defined as histopathological evidence of necrosis on resected bowel specimens (*n* = 11; 11 of 112; 9.8%). Reversible ischemia was defined as intraoperative evidence of impaired blood supply that resolved after the obstruction was lifted or the involved loops were placed in warm saline (*n* = 13; 13 of 112; 11.6%).

### CT examination

All patients underwent a multiphase contrast-enhanced DECT covering the abdomen and pelvis using a 160-mm 3rd generation rsDECT (Revolution^TM^ CT or Revolution^TM^ Apex or Revolution^TM^ Frontier; GE Healthcare). A TUE CT scan was first acquired (120 kVp, collimation 40 mm, pitch 1.375, rotation 0.7 s) within one breath hold. Raw data were reconstructed with a slice thickness/interval of 1.25/1.25.

A portal-venous phase DECT was then acquired 80 s after the intravenous injection of 1.5 cc/kg of non-ionic contrast medium (iomeprol, 350 mg iodine/mL; Iomeron 350, Bracco Imaging, or iobitridol, 350 mg iodine/mL, Xenetix 350, Guerbet) through a power injector at a rate of 2.5–3 mL/s. The portal-venous phase images were acquired in dual-energy mode with rapid kVp-switching between 80 and 140 kVp (tube current 610 mA, collimation 80 mm, pitch 0.5, rotation 0.6 s, noise index 20). Portal-venous phase-derived VUE images, iodine images, and monoenergetic images at 50 keV and 77 keV were reconstructed with a slice thickness/interval of 1.25/1.25. In agreement with the CT constructor recommendations, monoenergetic images at 77 keV were considered equivalent to conventional single-energy CT images obtained at 120 kVp. Adaptive Statistical Iterative Reconstruction (ASIR-V) was used until December 2020 at Henri Mondor University hospital and April 2022 at Paris-Saint-Joseph hospital (*n* = 80). Afterwards, a deep learning-based image reconstruction algorithm (DLIR, True Fidelity, GE Healthcare) was used (*n* = 32). Radiation exposure was investigated, and the dose-length product (DLP, mGy.cm) and volume CT dose index (CTDIvol, mGy) values of both TUE and portal-venous phase series were systematically collected.

### Readers visual assessment

**Fig. 2:**
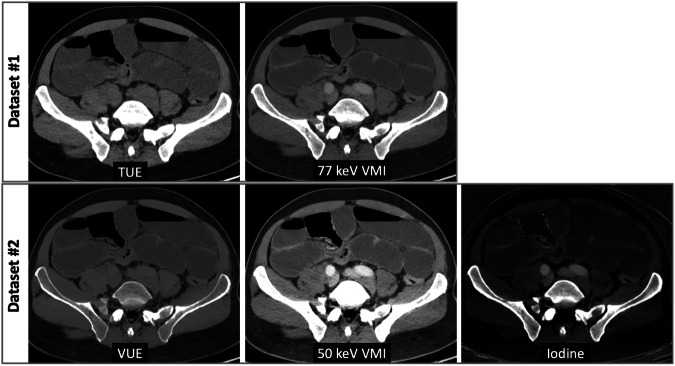
Image combination used for each dataset. The dataset#1 included the TUE images and the monoenergetic images at 77 keV, while the dataset #2 included the VUE images, the monoenergetic images at 50 keV, and the iodine images. Hence, datasets #1 and #2 corresponded to 120 kVp-equivalent polychromatic CT examination and DECT examination, respectively. TUE, true unenhanced; VUE, virtual unenhanced; VMI, virtual monoenergetic image

For each patient, two image datasets were considered. The dataset #1 included the TUE images and the monoenergetic images at 77 keV, while the dataset #2 included the VUE images, the monoenergetic images at 50 keV, and the iodine images. Hence, datasets #1 and #2 corresponded to 120 kVp-equivalent polychromatic CT examination and DECT examination, respectively (Fig. 2).

Datasets #1 and #2 were randomly and independently reviewed by three readers (two senior readers, S.M. and M.B., and one junior reader, B.B.) with 11, 7, and 4 years of experience in abdominal imaging, respectively. The three readers were aware of the diagnosis of mechanical SBO, but without knowledge of any clinical and surgical findings. Two reading sessions were organized with a wash-out time of 4 weeks between sessions to limit reader recall. For each patient, datasets #1 and #2 were randomly distributed between the two sessions so that 1) the order and the dataset number of the cases in each session were random, and 2) datasets from the same patient were not reviewed during the same reading session. For each dataset, the following three major features were visually evaluated: (a) reduced bowel wall enhancement, (b) a closed-loop mechanism defined by at least two adjacent abrupt transition zones on a segment of bowel along its course, and (c) diffuse mesenteric haziness. Diagnosis of bowel ischemia (including both reversible ischemia and necrosis) was defined at CT if at least two of these three CT findings were present [6]. Diagnosis of bowel necrosis (irreversible ischemia) was defined at CT if an increased bowel wall attenuation of a distended small-bowel loop was present on unenhanced images compared with the neighboring loops [7, 10]. In addition, those four features were evaluated regarding their individual sensitivity/specificity for determining bowel ischemia and necrosis, respectively.

The following features were also evaluated: (d) small-bowel wall thickening, defined as bowel wall measurement greater than 3 mm on loops located upstream of the transition zone, (e) feces sign, (f) free peritoneal gas, (g) intramural gas, (h) mesenteric venous and/or portal-venous gas.

Overall image quality was also visually evaluated using the following four-grade scale: 1 = non-diagnostic images; 2 = low image quality, but diagnostic images; 3 = good image quality with minor artifacts/noise; 4 = high quality without any impairment by artifact or noise.

### Semi-quantitative assessment

**Fig. 3:**
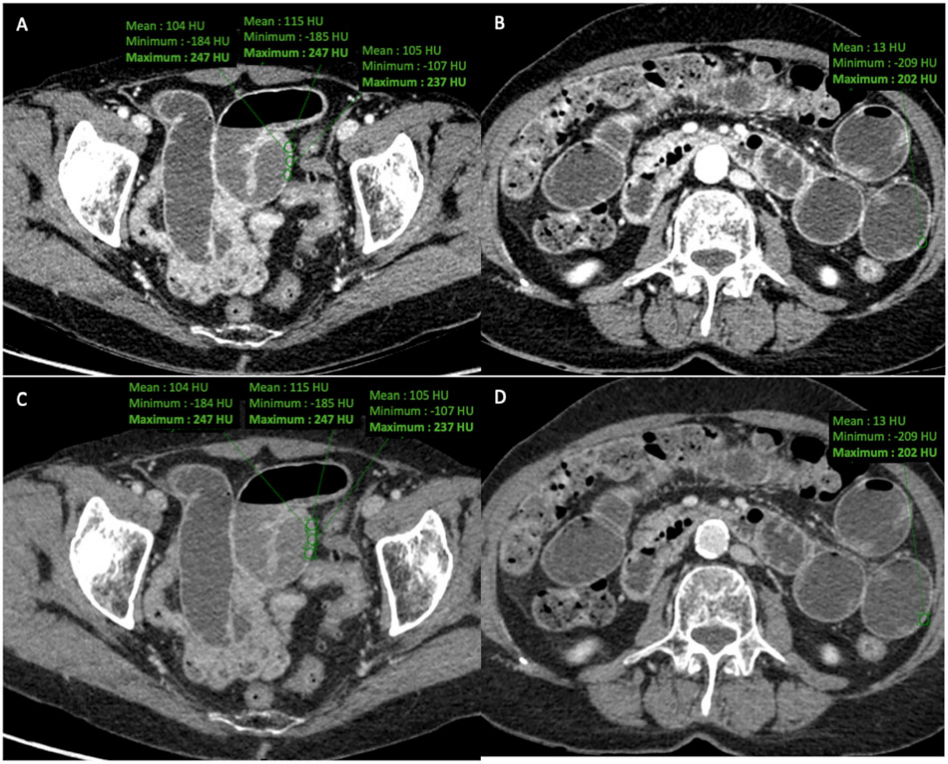
Quantitative assessment of attenuation and iodine concentration of bowel wall on monoenergetic images at (**A**, **B**) 50 keV or (**C**, **D**) 77 keV. Transmural region-of-interests (green circles) were placed across the wall of (**A**, **C**) the involved loop and of (**B**, **D**) a proximal dilated loop, respectively. The four ROIs were then duplicated on TUE, VUE, and iodine images. Maximum values were extracted from each ROI, and the values from the 3 ROIs placed on the involved loop were averaged

Two of the three readers involved in the visual analysis identified all the transition zones on monoenergetic images at 50 keV or 77 keV (during datasets #1 and #2 analysis, respectively) and manually placed three oval transmural region-of-interests (ROI) across the wall of the involved loop located upstream of the transition zone—or in the enclosed loop in case of closed-loop mechanism with more than one single transition zone (Fig. 3). An additional ROI was placed across the wall of a proximal dilated loop. The four ROIs were placed, avoiding adjacent structures and feces with higher attenuation than the bowel wall. They were then duplicated on other images for each set, i.e., on TUE images for dataset #1, and on VUE and iodine images for dataset #2. Finally, maximum values were extracted from each ROI, and the values from the 3 ROIs placed on the involved loop were averaged.

### Statistical analysis

Continuous variables are provided as median and interquartile range. Categorical variables as count and percentages. Chi-square and Fisher exact tests were used to compare frequencies of categoric variables between the two datasets, as appropriate. The paired sample t-test and Wilcoxon rank-sum test were performed for continuous variables, as appropriate.

The intrareader concordance between both datasets for each CT finding was calculated by using Cohen’s kappa statistics.

For each dataset, the sensitivity, specificity, and accuracy of each CT finding for bowel ischemia (including both reversible and irreversible ischemia) were estimated, along with their 95% confidence intervals. Both per-reader and pooled results were calculated. The performance of the iodine concentration for the prediction of bowel ischemia and bowel necrosis was evaluated using receiver operating characteristic (ROC) curve analysis. The optimal cutoff value corresponding to maximal Youden index was determined using a non-parametric approach. Associated sensitivity, specificity, and accuracy were provided with associated 95% CI.

The interreader agreement for each CT finding on both datasets was assessed by using Fleiss’s kappa statistics and was interpreted as follows: no agreement, 0–0.20; weak agreement, 0.21–0.40; moderate agreement, 0.41–0.60; good agreement, 0.61–0.80; and excellent agreement, 0.81–1.

Two-sided *p*-values < 0.05 were considered statistically significant. Statistical analyses were performed by using R software (v.3.4.1).

## Results

### Participant characteristics

A total of 112 patients met the inclusion criteria, including 64 women (57.1%; 64 of 112) and 48 men (42.9%; 48 of 112), with a median age of 72 years (interquartile range (IQR), 60–84 years). The median body-mass index was 22.7 (IQR, 19.9–26.2; range, 12.8–37.9). The main baseline demographic and clinico-pathologic characteristics of patients are detailed in Table 1.

**Table 1:** Baseline demographic characteristics and clinical and laboratory parameters of patients

Characteristic	All Patients (*n* = 112)	Patients with ischemia (*n* = 24)	Patients without ischemia (*n* = 88)	*p*-value^a^
Age	71.5 (59.8–84)	79 (60–87.5)	70 (59.8–82.3)	0.19
Sex, *n* (%)				0.82
Male	48 (42.9)	11 (45.8)	37 (42.0)	
Female	64 (57.1)	13 (54.2)	51 (58.0)	
Body-mass index, kg.m^-2^	23.3 ± 4.4	22.0 ± 3.6	23.7 ± 4.5	0.22
Serum creatinine level, µmol/L	76 (59.5–104)	74 (59.5–95.5)	77 (59.5–104.5)	0.66
White blood count, × 10^3^/μL	10.55 (8.7–14.1)	13.05 (10.5–14.6)	10.05 (8. 5–12.7)	0.017
C-reactive protein, mg/L	13 (4–50)	6 (4–32.7)	16 (4–52.5)	0.21

Except where indicated, data are medians, with interquartile ranges in parentheses

^a^ Statistical comparison between patients with bowel ischemia and patients without bowel ischemia

### Reference standard

Forty-one out of the 112 patients (36.6%) underwent surgical treatment, with a diagnosis of adhesive bands in 21 patients (51.2%; 21 of 41), external hernia in 9 patients (22.0%; 9 of 41), internal hernia in 4 patients (9.8%; 4 of 41), small-bowel volvulus in 3 patients (7.3%; 3 of 41), ileitis in two patients (4.9%; 2 of 41), small-bowel adenocarcinoma in one patient (2.4%; 1 of 41), and bezoar in one patient (2.4%; 1 of 41); seventy-one patients (63.4%) had non-surgical management without event during follow-up. The median delay between CT examination and surgery was one day (IQR, 0–1). Among the 41 patients who underwent surgery, 24 patients (58.5%; 24 of 41) had findings of ischemia, including 13 reversible cases (54.2%; 13 of 24) with spontaneous resolution after adhesive band section, and 11 irreversible cases (45.8%; 11 of 24) that required bowel resection. In those 11 patients, complete transmural necrosis was confirmed by pathological analysis. Median hospital stay after CT scan was 4 days (IQR: 3–9.25). Three patients (2.7%, 3 of 112) died from multivisceral failure during their hospital stay. In patients with conservative management, the median length of follow-up was 155 days (interquartile range, 12–403 days).

### Performance of each CT finding for the diagnosis of bowel ischemia and bowel necrosis

Overall image quality was high and similar in both datasets for the three readers (median, 4; IQR, 4–4 in dataset #1 vs. median, 4; IQR 4–4 in dataset #2 for the three readers; *p* = 0.35, *p* = 0.54 and *p* = 0.49 for readers #1, #2, and #3, respectively). Image quality of TUE and VUE images was not significantly different for the three readers (median, 4; IQR, 4–4 vs. median, 3; IQR 3–4 for the three readers; *p* = 0.22, *p* = 0.37, and *p* = 0.29 for readers #1, #2, and #3, respectively). Similarly, image quality of the monoenergetic images at 50 keV and 70 keV images was not significantly different for the three readers (median, 4; IQR, 4–4 vs. median, 4; IQR 4–4 for the three readers; *p* = 0.56, *p* = 0.47*,* and *p* = 0.69 for readers #1, #2, and #3, respectively). Finally, the image quality of the iodine images was high for the three readers (median, 4; IQR, 3–4 for readers #1 and #2; median, 4; IQR, 4–4 for reader #3).

**Fig. 4:**
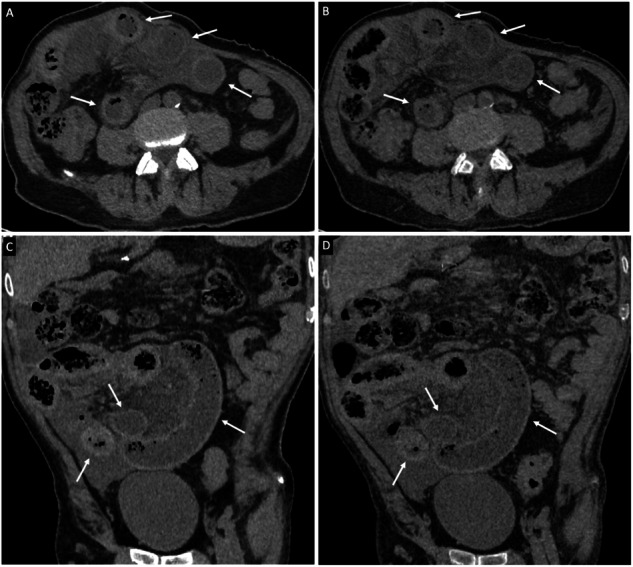
CT images in a 71-year-old man with small-bowel ischemia resulting from closed-loop small-bowel obstruction. The patient was treated by surgery and underwent bowel resection. (**A**, **B**) Axial and (**C**, **D**) coronal; (**A**, **C**) TUE and (**B**, **D**) VUE images show increased mural attenuation of the involved loops (arrows)

Increased bowel wall attenuation on unenhanced images showed similar diagnostic value on VUE or TUE images for the three readers, with 94.3–100% specificity and 37.5–50% sensitivity for diagnosis of bowel ischemia (Table 2), and 92.1–97% specificity and 45.5–63.6% sensitivity for diagnosis of bowel necrosis (Table 3) (Fig. 4).

**Table 2:** Summary diagnostic performance of each CT finding from both DECT (dataset #2) and 120 kVp-equivalent conventional CT (dataset #1) for the detection of bowel ischemia in patients with mechanical SBO

	Dataset #1				Dataset #2				*p*-value^a^			
	Reader #1	Reader #2	Reader #3	Pooled readers	Reader #1	Reader #2	Reader #3	Pooled readers	Reader #1	Reader #2	Reader #3	Pooled readers
Reduced bowel wall enhancement												
Sensitivity (%)	14/24 (58.3)	12/24 (50.0)	10/24 (41.7)	36/72 (50.0)	12/24 (50.0)	15/24 (62.5)	14/24 (58.3)	41/72 (56.9)	0.77	0.56	0.39	0.50
Specificity (%)	87/88 (98.9)	84/88 (95.5)	86/88 (97.7)	257/264 (97.3)	87/88 (98.9)	88/88 (100)	87/88 (98.9)	262/264 (99.2)	1	0.12	1	0.18
Closed-loop mechanism												
Sensitivity (%)	21/24 (87.5)	19/24 (79.2)	21/24 (87.5)	61/72 (84.7)	20/24 (83.3)	20/24 (83.3)	21/24 (87.5)	61/72 (84.7)	1	1	1	1
Specificity (%)	80/88 (90.9)	80/88 (90.9)	79/88 (89.8)	239/264 (90.5)	80/88 (90.9)	80/88 (90.9)	80/88 (90.9)	240/264 (90.9)	1	1	1	1
Diffuse mesenteric haziness												
Sensitivity (%)	20/24 (83.3)	20/24 (83.3)	19/24 (79.2)	59/72 (81.9)	22/24 (91.7)	20/24 (83.3)	19/24 (79.2)	61/72 (84.7)	0.67	0.86	1	0.82
Specificity, (%)	44/88 (50.0)	61/88 (69.3)	71/88 (80.7)	176/264 (66.7)	45/88 (48.9)	56/88 (63.6)	69/88 (78.4)	170/264 (64.4)	1	0.51	0.85	0.85
Increased unenhanced bowel wall attenuation												
Sensitivity (%)	12/24 (50.0)	10/24 (41.7)	10/24 (41.7)	32/72 (44.4)	9/24 (37.5)	9/24 (37.5)	10/24 (41.7)	28/72 (38.9)	0.56	1	1	0.61
Specificity (%)	87/88 (98.9)	88/88 (100)	88/88 (100)	263/264 (99.6)	83/88 (94.3)	88/88 (100)	88/88 (100)	259/264 (98.1)	0.21	1	1	0.22

^a^ Statistical comparison between Datasets #1 and #2

**Table 3:** Summary diagnostic performance of each CT finding from both DECT (dataset #2) and 120 kVp-equivalent conventional CT (dataset #1) for the detection of bowel necrosis in patients with mechanical SBO

	Dataset #1				Dataset #2				*p*-value^a^			
	Reader #1	Reader #2	Reader #3	Pooled readers	Reader #1	Reader #2	Reader #3	Pooled readers	Reader #1	Reader #2	Reader #3	Pooled readers
Reduced bowel wall enhancement												
Sensitivity (%)	8/11 (72.7)	8/11 (72.7)	8/11 (72.7)	24/33 (72.7)	8/11 (72.7)	8/11 (72.7)	9/11 (81.8)	25/33 (75.8)	1	1	1	1
Specificity (%)	94/101 (93.1)	92/101 (91.1)	97/101 (96.0)	283/303 (93.4)	96/101 (95.0)	94/101 (93.1)	95/101 (94.1)	285/303 (94.1)	0.77	1	0.75	0.87
Closed-loop mechanism												
Sensitivity (%)	11/11 (100)	11/11 (100)	11/11 (100)	33/33 (100)	11/11 (100)	11/11 (100)	11/11 (100)	33/33 (100)	1	1	1	1
Specificity (%)	81/101 (80.2)	84/101 (83.3)	82/101 (81.2)	247/303 (81.5)	81/101 (80.2)	83/101 (82.2)	83/101 (82.2)	247/303 (81.5)	1	1	1	1
Diffuse mesenteric haziness												
Sensitivity (%)	10/11 (90.9)	9/11 (81.8)	9/11 (81.8)	28/33 (84.8)	11/11 (100)	9/11 (81.8)	9/11 (81.8)	29/33 (87.9)	1	1	1	1
Specificity (%)	47/101 (46.5)	64/101 (63.4)	74/101 (73.3)	185/303 (61.1)	45/101 (44.6)	58/101 (57.4)	72/101 (71.3)	175/303 (57.8)	0.89	0.56	0.88	0.46
Increased unenhanced bowel wall attenuation												
Sensitivity (%)	7/11 (63.6)	6/11 (54.5)	7/11 (63.6)	20/33 (60.6)	6/11 (54.5)	5/11 (45.5)	7/11 (63.6)	18/33 (54.5)	1	1	1	0.80
Specificity (%)	95/101 (94.1)	95/101 (94.1)	98/101 (97.0)	288/303 (95.0)	93/101 (92.1)	95/101 (94.1)	98/101 (97.0)	286/303 (94.4)	0.78	1	1	0.86

^a^ Statistical comparison between Datasets #1 and #2

**Fig. 5:**
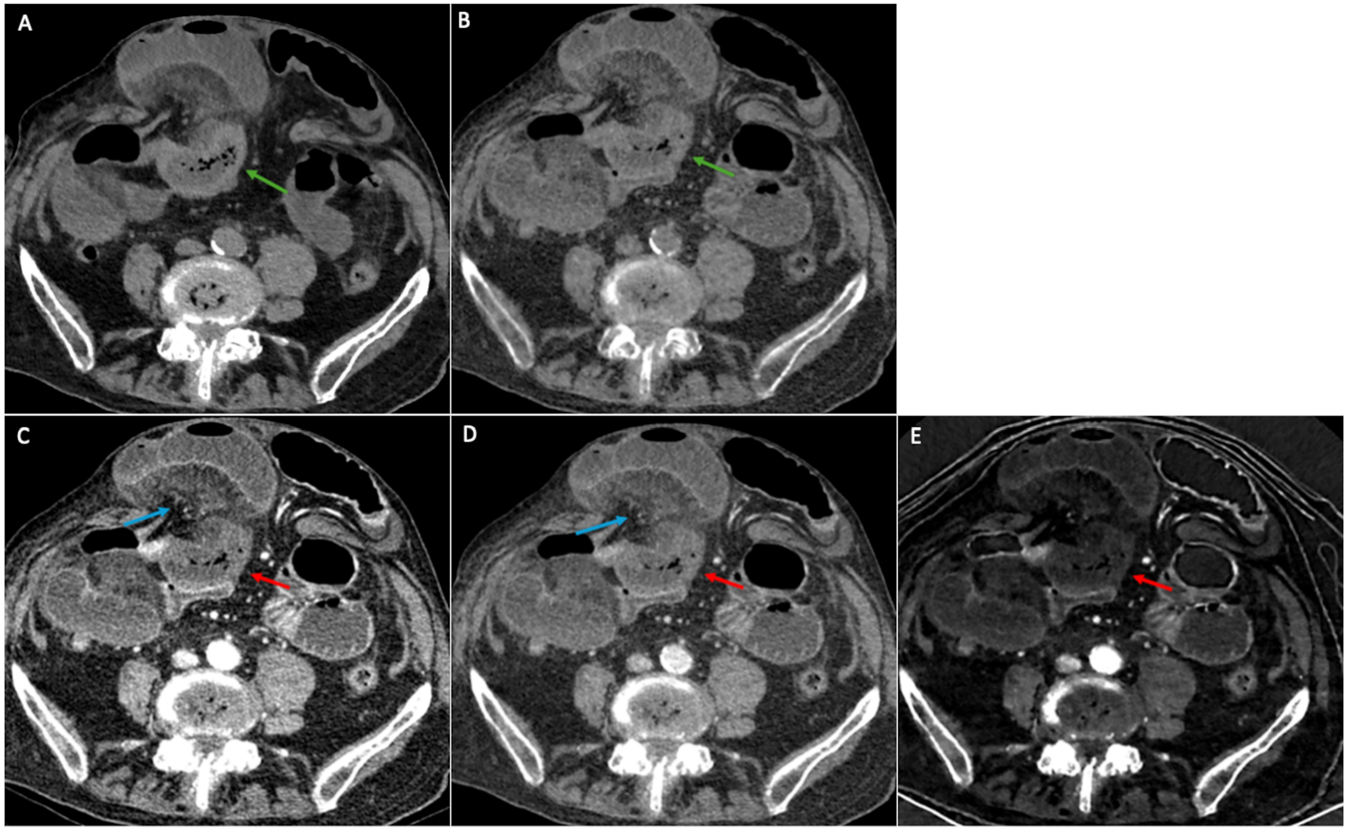
CT images in a 94-year-old woman with small-bowel ischemia secondary to incarceration within an umbilical eventration. The patient was treated by surgery and underwent bowel resection. Axial (**A**) TUE and (**B**) VUE images show increased bowel wall attenuation of the involved loop (green arrows). Axial monoenergetic images at (**C**) 50 keV and (**D**) 77 keV show a reduced bowel wall enhancement of the involved loop (red arrows) and presence of mesenteric haziness (blue arrows). **E** Iodine image also shows a reduced mural iodine uptake in the involved loop. The mural iodine concentration of the involved loop was 1.3 mg/mL

Diagnostic performance of reduced bowel wall enhancement, a closed-loop mechanism, and diffuse mesenteric haziness was also not statistically significant between both datasets for the three readers for the diagnosis of bowel ischemia and of bowel necrosis. When considering at least two out of 3 signs for the identification of both bowel ischemia and bowel necrosis, sensitivity and specificity values were identical on both datasets for all readers, respectively, higher than 75% and 80% (Table 4 and Fig. 5).

**Table 4:** Global diagnostic performance of DECT (dataset #2) in comparison with 120 kVp-equivalent conventional CT (dataset #1) for the detection of bowel ischemia and bowel necrosis in patients with SBO

	Dataset #1				Dataset #2				*p*-value^a^			
	Reader #1	Reader #2	Reader #3	Pooled readers	Reader #1	Reader #2	Reader #3	Pooled readers	Reader #1	Reader #2	Reader #3	Pooled readers
Bowel ischemia												
Sensitivity (%)	22/24 (91.7)	17/24 (70.8)	17/24 (70.8)	56/72 (77.8)	21/24 (87.5)	19/24 (79.2)	20/24 (83.3)	60/72 (83.3)	1	0.74	0.49	0.53
Specificity (%)	83/88 (94.3)	86/88 (97.7)	86/88 (97.7)	255/264 (96.6)	83/88 (94.3)	88/88 (100)	87/88 (98.9)	258/264 (97.7)	1	0.50	0.50	0.60
Bowel necrosis												
Sensitivity (%)	11/11 (100.0)	10/11 (90.9)	10/11 (90.9)	31/33 (93.9)	11/11 (100.0)	10/11 (90.9)	11/11 (100.0)	32/33 (97.0)	1	1	1	1
Specificity (%)	85/101 (84.2)	92/101 (91.1)	92/101 (91.1)	269/303 (88.8)	86/101 (85.1)	92/101 (91.1)	91/101 (90.1)	269/303 (88.8)	1	1	1	1

^a^ Statistical comparison between Datasets #1 and #2

### Intra- and interreader agreement between DECT and conventional CT datasets

All intra- and interreader agreement values of all CT findings are detailed in Table S1. Intrareader agreement between DECT and conventional CT datasets was excellent for depicting increased bowel wall attenuation and a closed-loop mechanism for all three readers. Intrareader agreement was good to excellent for reduced bowel wall enhancement, while it was highly variable (from moderate to excellent) among readers for diffuse mesenteric haziness.

Interreader agreement values were highly variable among CT findings but not statistically different between the two datasets. Findings with excellent interreader agreement values were reduced bowel wall enhancement, closed-loop mechanism, feces sign, free peritoneal gas, and mesenteric venous and/or portal-venous gas, while interreader agreement values associated with intramural gas were found to be low.

### Quantitative analysis

Mural attenuation and relative iodine concentration values of both involved loop and proximal dilated loop are summarized in Table 5.

**Table 5:** Mural attenuation and relative iodine concentration values of both involved and proximal dilated bowel loops in patients without bowel ischemia, with bowel ischemia (including reversible ischemia and necrosis), and in the subgroup of patients with bowel necrosis

	Patients without bowel ischemia (*n* = 88)			Patients with bowel ischemia (*n* = 24)			Patients with bowel necrosis (*n* = 11)		
	Proximal dilated loops	Involved loops	*p*-value	Proximal dilated loops	Involved loops	*p*-value	Proximal dilated loops	Involved loops	*p*-value
Dataset #1									
TUE (HU)	46.7 ± 10.7	52.0 ± 11.1	0.0025	48.2 ± 14.7	59.1 ± 15.8	0.025	52.1 ± 11.4	62.9 ± 15.5	0.11
77 keV-VMI (HU)	80.9 ± 22.0	86.9 ± 22.4	0.02	86.0 ± 28.7	77.3 ± 21.4	0.28	89.6 ± 31.4	78.3 ± 20.2	0.55
Dataset #2									
VUE (HU)	47.9 ± 13.6	50.7 ± 12.5	0.14	46.9 ± 13.3	57.5 ± 13.5	0.025	48.4 ± 14.0	57.7 ± 13.4	0.22
50 keV-VMI (HU)	171.4 ± 48.2	176.6 ± 41.3	0.39	155.5 ± 50.8	141.8 ± 51.9	0.26	179.0 ± 48.2	134.9 ± 47.0	0.022
Iodine concentration (mg/mL)	2.4 ± 0.7	2.5 ± 0.6	0.22	2.1 ± 0.7	1.7 ± 0.8	0.021	2.5 ± 0.6	1.5 ± 0.8	0.0031

Bowel wall attenuation of involved loops was significantly higher than that of proximal dilated loops on both TUE and VUE in patients with bowel ischemia. However, the difference was not significant in the subgroup of patients with bowel necrosis. Bowel wall attenuation values on VMI at 50 keV and 77 keV and relative iodine concentration values of involved loops were significantly lower in patients with ischemia compared to patients without ischemia, the difference being more pronounced when on VMI at 50 keV (141.8 ± 51.9 and 176.6 ± 41.3, *p* = 0.0008). Bowel wall attenuation of the involved loops was significantly higher in patients with bowel necrosis than in patients without necrosis on TUE images (62.9 ± 15.5 and 52.5 ± 11.8, *p* = 0.035), while the difference was not statistically significant on VUE images (57.7 ± 13.4 and 51.6 ± 12.8, *p* = 0.19). In patients with bowel necrosis, bowel wall attenuation values on VMI at 50 keV and relative iodine concentration values of the involved loops (134.9 ± 47.0 and 1.5 ± 0.8, respectively) were significantly lower than that of proximal dilated loops (179.0 ± 48.2, *p* = 0.022 and 2.5 ± 0.6, *p* = 0.0031, respectively) and also than that of involved loops in patients without necrosis (172.8 ± 44.4, *p* = 0.0084 and 2.4 ± 0.7, *p* = 0.0073, respectively).

Quantitative ROC curve analyses showed that relative iodine concentration images confidently identify bowel ischemia and bowel necrosis. Indeed, the AUROC for the identification of bowel ischemia with relative iodine concentration was 0.81 (95% CI 0.78, 0.98). With an optimal cutoff value of 1.85 mg/mL, sensitivity and specificity were 84% and 73%, respectively. Regarding bowel necrosis, the AUROC with relative iodine concentration was 0.86 (95% CI 0.78, 0.98). With an optimal cutoff value of 1.82 mg/mL, sensitivity and specificity were 82% and 83%, respectively.

### Radiation dose

The specific mean DLP of TUE and portal-venous phase DECT images was 441.6 ± 204.3 mGy.cm and 602.2 ± 112.3 mGy.cm, respectively. The mean CTDI_vol_ of TUE and portal-venous phase DECT images were 8.23 ± 2.51 mGy and 9.77 ± 2.48 mGy, respectively, accounting for 41% and 59% of the total CTDI_vol_, respectively.

## Discussion

Our study demonstrates the ability of DECT to detect bowel ischemia in patients with mechanical SBO. The performance of DECT for depicting ischemia was similar to that of 120 kVp-equivalent CT. In addition, our results highlight the potential interest in the semi-quantitative analysis of bowel wall iodine concentration to identify ischemic and necrotic bowel loops and to impact clinical decision-making by identifying patients requiring surgery.

Prompt identification of ischemia in patients with mechanical SBO is as crucial as it is challenging and does not rely on a single imaging feature but rather on the combination of several imaging features [6, 8, 16]. Hence, a closed-loop mechanism, decreased bowel wall enhancement, and diffuse mesenteric haziness are CT features predictive of ischemia according to the several meta-analyzes [3, 17, 18], while increased unenhanced bowel wall attenuation is predictive for bowel necrosis with high specificity, requiring immediate surgical intervention in adhesive SBO [7, 9, 19, 20]. In our study, when considering at least two out of 3 signs for the identification of both bowel ischemia and bowel necrosis, sensitivity and specificity values were higher than 70% and 84%, respectively, on both datasets for all readers. This is in line with previous results on conventional contrast-enhanced CT [3, 8, 11]. The good performance of conventional CT may explain the challenge of demonstrating the added value of DECT and explain the lack of significant difference between the two CT techniques in our study. Nonetheless, our results show that DECT is at least non-inferior to conventional CT for the identification of bowel ischemia in patients with mechanical SBO.

A closed-loop mechanism is a CT feature associated with a high risk of ischemia [21]. In our study, the incidence of closed-loop obstruction was 27.7% (31 of 112 patients), in agreement with previous studies [6, 7, 21]. Among these 31 cases, bowel ischemia was found in 22 patients (71.0%), while the 9 other patients (29%) had a favorable outcome with conservative management only. This is in agreement with Rondenet et al, who reported a 31% rate of favorable outcome in patients with closed-loop SBO who received first-line conservative treatment [22].

Evaluation of bowel wall enhancement is of paramount importance in SBO but remains subjective with significant interreader variability [3, 12, 23]. We found similar interreader agreement values when reading DECT images or 120 kVp-equivalent CT images, suggesting that the improved image contrast on low-keV monoenergetic images was not hampered by associated increased noise. However, Darras et al found that optimal contrast-to-noise ratio values for small-intestinal mural enhancement were observed on VMI at 70 keV using a dual-source dual-energy CT platform [24]. The increasingly widespread use of deep learning-based image reconstructions may further improve the diagnostic value of low-keV monoenergetic images for decreased bowel wall enhancement [25, 26]. Quantitative analysis of bowel wall enhancement may also improve the diagnostic performance of DECT. Hence, Lourenco et al found lower attenuation values in ischemic loops compared to non-ischemic loops on single-energy CT images, the difference being more pronounced on 40 keV monoenergetic images [13]. They also highlighted significantly lower iodine uptake in ischemic bowel loops compared to non-ischemic loops. In our study, the relative iodine concentration within the obstructed bowel wall allows for the identification of ischemia and necrosis with almost perfect specificity and good sensitivity. As the added value of qualitative analysis of iodine images remains unclear [15], our results support the high interest in the quantitative analysis of iodine mapping for the diagnosis of bowel ischemia in patients with mechanical SBO.

We found that VUE and TUE images had no significantly different performances for the visual identification of increased bowel wall attenuation, with sensitivity and specificity values for bowel ischemia and bowel necrosis in agreement with those reported in the literature [8]. Indeed, the presence of increased bowel wall attenuation on both VUE and TUE images was highly specific for bowel ischemia and bowel necrosis, with low to moderate sensitivity. However, interreader agreement was lower in the interpretation of VUE images than TUE images. Quantitative analysis showed that, unlike TUE images, the difference in bowel wall attenuation values on VUE images of the involved loops between patients with and without bowel necrosis was not statistically significant. Although the absence of statistical significance may be due to a lack of statistical power, these results—in addition to the lower interreader agreement with VUE images than with TUE images—may suggest that analysis of VUE images may be more challenging than that of TUE images. Nevertheless, our results could justify not carrying out TUE image acquisitions and thus could represent a step forward towards radiation dose reduction. One may argue that unenhanced images also allow the analysis of the bowel at two different times (before injection and at the portal-venous phase), which may prevent misdiagnosis of false transition zones due to peristalsis. In that case, the acquisition of arterial phase images rather than unenhanced images may be preferred, as arterial phase images may help in identifying other causes of acute abdomen, including acute arterial thromboembolic occlusion and non-occlusive mesenteric ischemia [27]. In patients without bowel wall ischemia, a small but significant difference in TUE values was found between dilated and involved loops. This unexpected result underlines the added value rather than the surrogate value of quantitative analysis over visual analysis. This is in line with recent results from Lamant et al, which underline that quantitative analysis of iodine concentration alone did not outperform a global visual analysis of conventional CT images [28].

Attenuation and iodine values within the bowel wall were evaluated by using the maximum value of transmural ROIs placed across the wall of both the involved and the proximal dilated loops, in agreement with previous studies [8, 16]. ROIs were placed, avoiding adjacent structures and feces with higher attenuation than the bowel wall. The maximum value within the ROI could thus reflect the objective attenuation/iodine content of the intestinal wall.

The choice of our reference standard for the diagnosis of bowel ischemia and necrosis at CT was based on previous studies. Indeed, Millet et al found that the combination of a reduced bowel wall enhancement, a closed-loop mechanism, and diffuse mesenteric haziness was the most effective for the identification of ischemia in adhesive SBO [6]. Among these findings, a meta-analysis demonstrated the high specificity of reduced bowel wall enhancement for the diagnosis of bowel ischemia [3]. Moreover, Rondenet et al identified increased unenhanced bowel wall attenuation as the only sign specific for bowel necrosis [10].

Clinical and laboratory variables were not significantly different between patients with and without bowel ischemia except from white blood count which was significantly higher in patients with ischemia. This result is in agreement with the review of Eze et al that found white blood count to be strongly and positively associated with ischemia at surgery [17].

Some limitations should be noted. First, this is a retrospective study, which may cause selection bias. The number of patients with bowel ischemia was relatively small, which could have underpowered the statistical analysis. In addition, the average BMI was low compared to that in other countries, which could potentially bias our results. However, this is one of the largest cohorts of patients with mechanical SBO investigated with contrast-enhanced DECT to date. Moreover, two centers with different patient recruitment were involved, which may reinforce the external validity of our results. Second, all CT examinations were performed using fast kV-switching DECT platforms, and it may be difficult to expand our results to other DECT technologies. Third, the reconstruction algorithm was not identical for all CT examinations, as a deep learning-based image reconstruction algorithm (DLIR, True Fidelity, GE Healthcare) was introduced and systematically used instead of Adaptive Statistical Iterative Reconstruction (ASIR-V) from December 2020 and April 2022 in both centers, respectively. Fourth, the maximum values extracted from ROIs placed in the involved loop are prone to artifacts/outliers, although the values from the 3 ROIs were averaged. Finally, the diagnostic performance of iodine concentration was evaluated on the same data as those used to define the threshold values, making these estimates likely optimistic. Moreover, iodine concentration values may be influenced by the reconstruction algorithm used. Further multiplatform external validation of the identified iodine thresholds would be required.

In conclusion, our study demonstrates that DECT shares similar performance with that of 120 kVp-equivalent CT for the diagnosis of bowel ischemia in patients with mechanical SBO. VUE images offer a viable alternative to TUE images for the identification of increased bowel wall attenuation in abdominal CECT performed in the acute setting. Furthermore, bowel wall relative iodine concentration may act as a quantitative biomarker for bowel ischemia and necrosis, and thus impact management strategy by identifying patients likely to require surgery.

## Supplementary information

ELECTRONIC SUPPLEMENTARY MATERIAL

## Abbreviations

CECT: Contrast-enhanced multidetector CT
CTDI: CT dose index
DECT: Dual-energy CT
ROC: Receiver operating characteristic
ROI: region-of-interest
SBO: Small-bowel obstruction
TUE: True unenhanced
VMI: Virtual monoenergetic images
VUE: Virtual unenhanced
