# Supplementary material for: Value of dual-energy computed tomography in the diagnosis of bowel ischemia in patients with mechanical small-bowel obstruction: a retrospective, dual-center study
Source: Eur Radiol. 2025 May 6;35(11):7320–32. doi: 10.1007/s00330-025-11635-9 (PMC12559072; doi:10.1007/s00330-025-11635-9)
Supplement: Supplementary file 1 — ELECTRONIC SUPPLEMENTARY MATERIAL [file 330_2025_11635_MOESM1_ESM.pdf]

Value of Dual-Energy Computed Tomography in the Diagnosis of Bowel ischemia in Patients with Mechanical Small Bowel Obstruction: a Retrospective, Dual-Center Study

ELECTRONIC SUPPLEMENTARY MATERIAL

Table S1: Intra- and interreader agreement between DECT and 120 kVp-equivalent conventional CT datasets for each CT finding

	Intrareader agreement between Datasets			Interreader agreement	
	Reader #1	Reader #2	Reader #3	Dataset #1	Dataset #2
Increased unenhanced bowel wall attenuation	0.85	0.83	0.89	0.71	0.41
Reduced bowel wall enhancement	0.83	0.77	0.79	0.81	0.79
Diffuse mesenteric haziness	0.46	0.70	0.84	0.42	0.43
Closed-loop mechanism	0.96	0.93	0.93	0.77	0.80
Small bowel wall thickening	0.60	0.84	0.81	0.76	0.71
Feces sign	0.78	0.91	0.94	0.80	0.82
Free peritoneal gas	1.00	0.80	0.85	0.81	0.76
Intramural gas	1.00	0.66	1.00	0.33	0.33
Mesenteric venous and/or portal venous gas	1.00	1.00	1.00	1.00	1.00
Diagnosis of ischemia	0.84	0.85	0.88	0.73	0.70
